# Perceived impacts of COVID-19 responses on routine health service delivery in Liberia and UK: cross-country lessons for resilient health systems for equitable service delivery during pandemics

**DOI:** 10.1186/s12913-023-09162-8

**Published:** 2023-03-29

**Authors:** Yussif Alhassan, Zeela Zaizay, Laura Dean, Rosalind McCollum, Victoria Watson, Karsor Kollie, Helen Piotrowski, Olivia Hastie, Colleen Parker, Russell Dacombe, Sally Theobald, Miriam Taegtmeyer

**Affiliations:** 1 Department of International Public Health, Liverpool School of Tropical Medicine, Pembroke Place, Liverpool, L3 5QA UK; 2 Actions Transforming Lives, Monrovia, Liberia; 3 Ministry of Health, Monrovia, Liberia; 4 UK Health Security Agency, London, UK

**Keywords:** COVID-19, Impact, Equity, Quality, Health services, Health system resilience, Liberia, Merseyside UK

## Abstract

**Background:**

COVID-19 has caused significant public health problems globally, with catastrophic impacts on health systems. This study explored the adaptations to health services in Liberia and Merseyside UK at the beginning of the COVID-19 pandemic (January–May 2020) and their perceived impact on routine service delivery. During this period, transmission routes and treatment pathways were as yet unknown, public fear and health care worker fear was high and death rates among vulnerable hospitalised patients were high. We aimed to identify cross-context lessons for building more resilient health systems during a pandemic response.

**Methods:**

The study employed a cross-sectional qualitative design with a collective case study approach involving simultaneous comparison of COVID-19 response experiences in Liberia and Merseyside. Between June and September 2020, we conducted semi-structured interviews with 66 health system actors purposively selected across different levels of the health system. Participants included national and county decision-makers in Liberia, frontline health workers and regional and hospital decision-makers in Merseyside UK. Data were analysed thematically in NVivo 12 software.

**Results:**

There were mixed impacts on routine services in both settings. Major adverse impacts included diminished availability and utilisation of critical health services for socially vulnerable populations, linked with reallocation of health service resources for COVID-19 care, and use of virtual medical consultation in Merseyside. Routine service delivery during the pandemic was hampered by a lack of clear communication, centralised planning, and limited local autonomy. Across both settings, cross-sectoral collaboration, community-based service delivery, virtual consultations, community engagement, culturally sensitive messaging, and local autonomy in response planning facilitated delivery of essential services.

**Conclusion:**

Our findings can inform response planning to assure optimal delivery of essential routine health services during the early phases of public health emergencies. Pandemic responses should prioritise early preparedness, with investment in the health systems building blocks including staff training and PPE stocks, address both pre-existing and pandemic-related structural barriers to care, inclusive and participatory decision-making, strong community engagement, and effective and sensitive communication. Multisectoral collaboration and inclusive leadership are essential.

**Supplementary Information:**

The online version contains supplementary material available at 10.1186/s12913-023-09162-8.

## Background

COVID-19 has caused a global public health emergency with a catastrophic impact on many aspects of society, economies and health [1]. Its effect on health systems has been particularly prominent: disrupting essential health services, exposing severe gaps in public health infrastructure [2] and threatening their resilience [3]. As of January 2023, around 660 million confirmed cases and 6.6 million deaths related to COVID-19 have been reported globally [4]. The COVID-19 response globally has been inequitable [5] and even well-resourced health systems have struggled to cope. Meanwhile, infection rates have continued to be high [4], with new variants of the virus emerging, the global economy is reeling which is threatening much needed investment in health systems in many countries.

Early health system responses to the COVID-19 pandemic focused on containing the spread of the virus and reducing related morbidity and mortality [6]. With the lack of specific therapies and vaccines, early responses were based on enhanced surveillance and a combination of different non-pharmaceutical interventions (NPIs), such as case quarantine, voluntary isolation, community lockdown, hand and environmental hygiene, and physical distancing, which proved successful in limiting the spread of the virus in most places. Cascini and colleagues underscored differential impact of NPI measures on transmission of COVID-19 in five European countries (France, Germany, Italy Spain and UK), and noted containment in the UK was limited by early relaxation of restrictive measures such as lockdown and wearing of face coverings [7]. In the UK, additional measures targeting the health services were introduced, including the freeing up available resources for the management of COVID-19 patients and the rapid adoption of telemedicine in primary and secondary care [8]. These brought dramatic changes to health service delivery and use [9]. In Liberia, drawing on her experience with the 2014–15 Ebola Virus Disease (EVD) epidemic, the government moved early to implement infection control measures, including lockdown, contact-tracing, social distancing, screening of travellers at ports of entry, and extensive awareness raising to address fear and stigma [10]. There was greater emphasis on the continuity of routine essential health services [5].

Health system measures adopted as part of COVID-19 response may have affected health service delivery both positively and negatively [11]. The pandemic has exposed fault lines in quality and equity access to non-COVID-care and higher rates of COVID-19 related infections and deaths in certain populations [12, 13] Patients with ongoing care needs, including pregnant women needing maternal health care; patients with chronic disease and patients needing chronic cancer care, have been disproportionately affected by disruptions in routine services resulting from the pandemic [14]. A study involving eight sub-Saharan African countries (Cameroon, Democratic Republic of Congo, Liberia, Malawi, Mali, Nigeria, Sierra Leone and Somalia) found a cumulative shortfall of 5,149,491 outpatient consultations for ante- and postnatal care and 328,961 missed third-dose pentavalent vaccinations during the first 5 months of the COVID-19 pandemic [15]. In Ethiopia, reduction in ante- and postnatal care utilisation was linked to pregnant women’s anxiety about COVID-19 infection and restrictions by government mitigation measures [14, 15]. In Mozambique, Bliznashka, Ahun [16] discovered disruptions to child health services linked with facility-based COVID-19 risk mitigation measures and financial inability to pay for personal protective equipment (PPE) which prevented caregivers from attending child health visits. Reports in the UK indicated high rates of cardiovascular deaths during the first wave of COVID-19 (January – May 2020) related to patient reluctance to seek care due to fear of hospital infection of COVID-19 and early government messaging for people to stay at home [17]; admissions to hospital Emergency Department for heart attacks fell by 50% in England between March – May 2020 [18]. Telephone consultation, widely adopted during the pandemic, has been associated with low service utilisation among elderly and low-income households and compromised patient safety [19]. Thomas, Sagan [3] argued that COVID-19 may have had an indirect impact on health service utilisation through its economic consequences, especially among people working in the informal sector, resulting in diminished ability to afford healthcare.

Although there has been extensive analyses of the public health impact of COVID-19 response measures, including effects on disease containment [7, 20], little is currently known about what consequences these health system measures may have had on non-COVID-19 (‘routine’) health services. Furthermore, with varying degrees of success in countries’ response to the pandemic, questions are being asked about what can be learnt from the response to help prepare for, and cope with, future waves of the current and future pandemics [11, 20, 21]. This study sought to describe the adaptations to health services in Liberia and Merseyside UK at the beginning of the COVID-19 pandemic (January–May 2020) and their perceived impact on the delivery of routine health services. Based on the perspectives of key actors at different levels of the health system, we aimed to identify cross-context lessons for building more equitable health systems during a pandemic response. Data from this study may inform response measures to minimise disruptions to essential health service delivery in public health emergencies.

## Methods

### Study design and conceptual framework

**Fig. 1:**
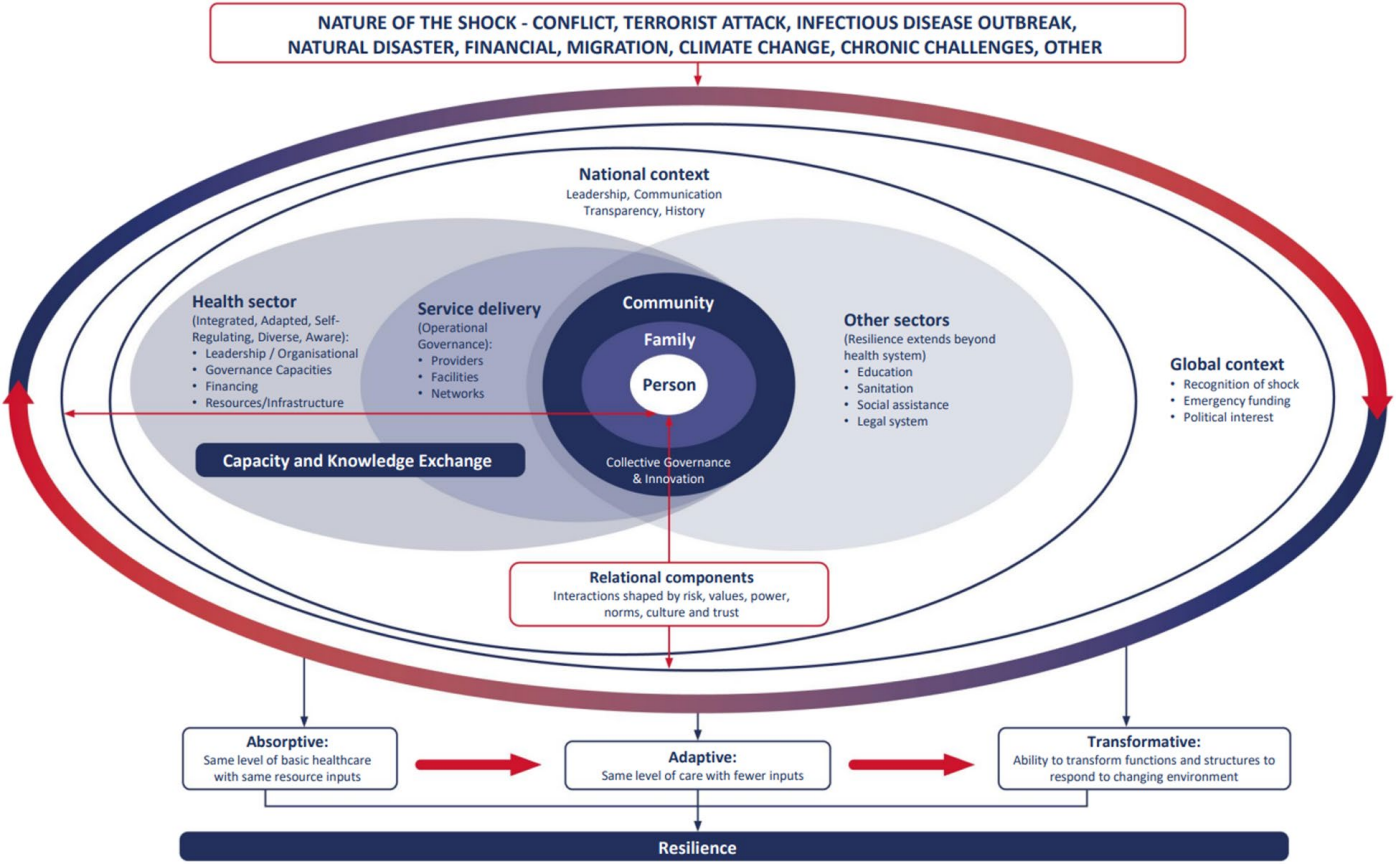
Conceptual framework: people-centred approach to health system resilience [25]

We employed a cross-sectional qualitative design to better understand COVID-19 adaptations and their impact on health services. This approach provides potential for exploring participant perspectives within their embedded context [22]. A collective case study approach involving simultaneous exploration and comparison of the experiences in Liberia and in Merseyside, UK was adopted to gain perspectives on health system responses in both low- and high-income contexts [23, 24]. The study was guided by a people-centred health systems resilience framework [25], adapted from existing resilience models [26–28] (Fig. 1). This framework places the person at the centre of a health systems response to a health shock (e.g., a pandemic), recognising their role within the health system as a beneficiary and a participant, and the need for response measures to be oriented to meeting their needs and maximising their wellbeing. The framework emphasises community and stakeholder engagement. It recognises the significance of the interdependencies of different sectors within the health system and between the health system and other systems to develop and implement a holistic pandemic response. Another key element of the framework is the importance and recognition of the health systems building blocks, and their ability to flex and respond resiliently to shock [29]. The model was used to both inform the design of topic guides and to frame the recommendations. The main questions examined were: What were the early COVID-19 health system response measures in Liberia and Merseyside (UK), and how were decisions about these made? What are health system actors’ perceptions of the impact of these adaptations on equity and quality of routine health care delivery? What cross-context learning may support the strengthening of health systems to maintain essential service delivery during times of crisis?

### Study population and recruitment

Data were collected from participants in Liberia and Merseyside, UK. These settings were selected due to demand from stakeholders for research to help inform their COVID-19 responses. The research team had strong pre-existing links and networks within both settings which facilitated timely access to data and sharing of study findings. The two contexts are different in terms of their level of economic development and health system organisation, but provide an opportunity to compare COVID-19 responses from low- and high-income settings and for the countries to learn from each other. A purposive sampling technique was employed to select participants in both contexts. Based on this approach, individuals were selected based on their role in the health system along with their ability and willingness to provide useful information relevant data [30]. This enabled us to obtain rich and sufficient information to address the research questions [22]. A total of 66 participants were purposively selected for the interviews because of their direct involvement with COVID-19 planning and/or routine service delivery during the pandemic. In Liberia, 24 participants were purposively selected from both county (Nimba, Margibi and Montserrado Counties) and national levels of the health system, including senior officials from relevant service areas such as health promotion, family health, noncommunicable diseases, community health laboratory and transfusion programmes. They all had decision making capacity in shaping the response within the Liberian health system. Some had been involved in the EVD epidemic response and allowed for reflections on how learning from the epidemic had informed the pandemic response. In Merseyside, 42 participants were selected, and included regional, hospital and primary care decision-makers (general practitioners and residential care home managers) and front-line health workers (Table 1). More participants were selected across different health systems levels in Merseyside due to demand for research across multiple levels in the former compared to Liberia where demand for research mainly focused at national level. Across both contexts, participants were identified through the networks of individual members of the two research teams as well as referral from interviewees. Sample size in each setting was determined by data saturation, deemed to have been reached when no new themes emerged at debriefing meetings with the research teams [31].

**Table 1:** Socio-demographic information of study participants

Participant role	Number Interviewed
**Liberia**	
National decision-makers	21
County decision-makers	3
**Total**	**24**
**Merseyside UK**	
Regional decision-makers	5
Hospital decision-makers (clinical director, medical director, ward manager)	4
Hospital consultants	11
Hospital health workers (junior doctors, nurses)	10
Health workers in community (GP, district nurse, residential care home managers)	7
Hospital laboratory staff	5
**Total**	**42**

### Data collection

Data in both study settings were collected by experienced researchers between June and September 2020. This occurred during the early stages of the pandemic when response measures in both contexts were being newly implemented, providing participants with the opportunity to reflect on their impact. A relative lull after the first wave of the pandemic afforded healthcare workers and policy actors the time and space to reflect and be interviewed about their experiences. Data were collected through individual interviews. Interviews were predominantly carried out remotely by experienced researchers in English language, via online platforms such as Microsoft Teams and Zoom due to physical distancing measures adopted to promote infection control. A minority were carried out in person with physical distancing measures, according to local guidance. Each interview lasted approximately 45 minutes and was audio-recorded. Topic guides were developed with broad open-ended questions based on the conceptual framework and explored key areas such as governance and decision-making; use of ethical guidelines; human resource management; infrastructure (information technology and communications) and health care worker support; introduction of innovations; and perceptions of the equity and quality of service delivery. Adaptations were made according to the health systems context in each country. While discussion in Merseyside focused at the regional and health facility levels, in Liberia, it focused on the national and county levels. Participants were interviewed once within the study timeframe. They were informed about the broad interview topics during the consent process and only heard about the specific questions during the interview.

### Data analysis

**Fig. 2:**
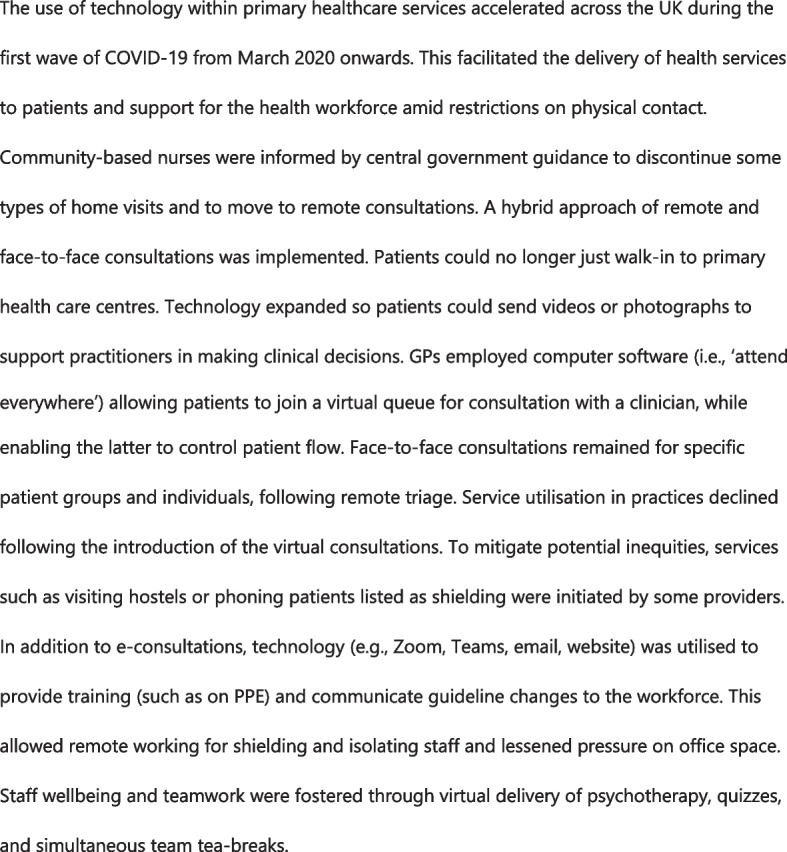
The role of technology in primary healthcare in Merseyside during COVID-19

**Fig. 3:**
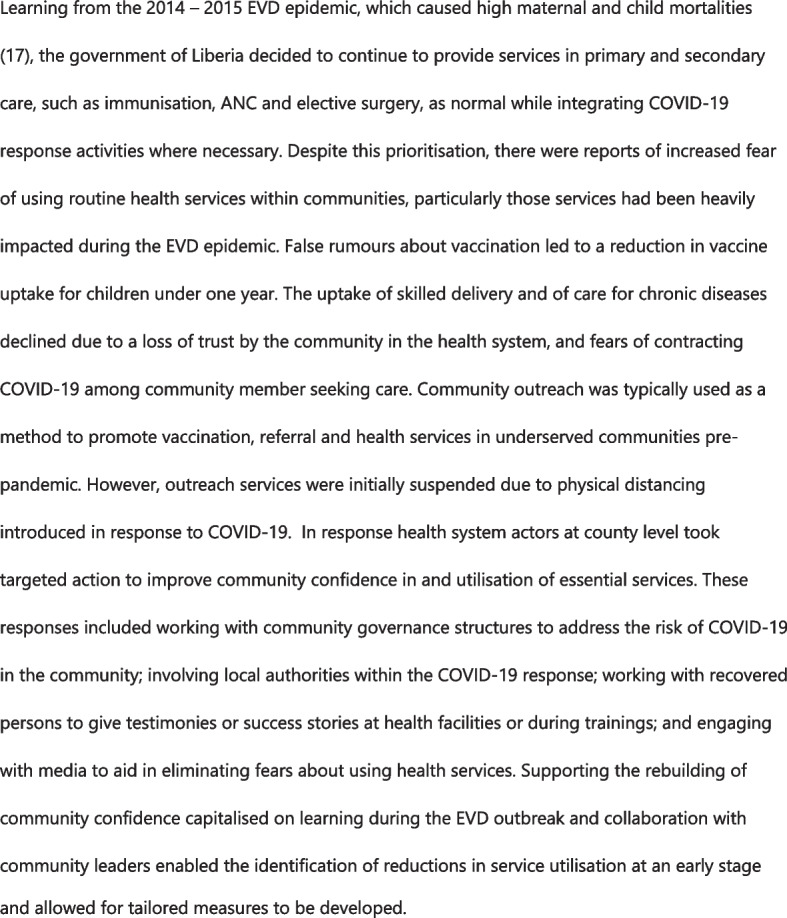
Community engagement to promote continued use of maternal and child health services in Liberia

**Fig. 4:**
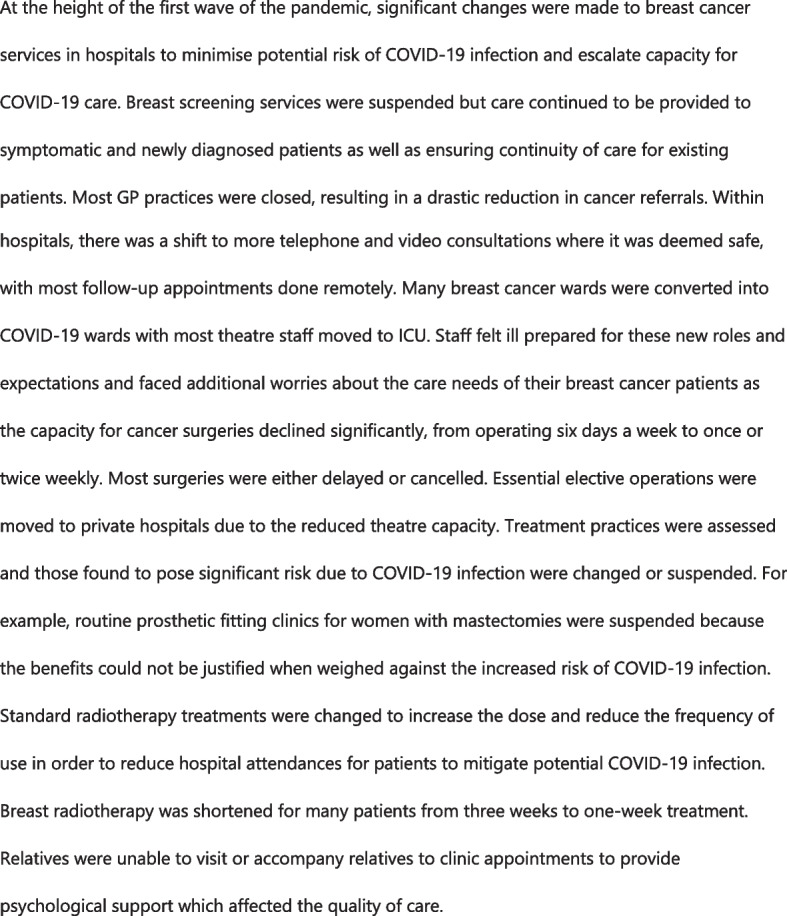
Disruptions in breast cancer services during COVID-19 in Merseyside

Audio-recordings were transcribed verbatim using Otter.ai software, with quality assurance conducted by a second researcher against the recording. Data were analysed with the support of NVivo12 software based on a framework analysis approach [32]. A coding framework was inductively developed for each of the contexts (based on the study data and the conceptual framework) by researchers involved in data collection. Data relating to individual components of the research question for each of the contexts were first analysed using the coding frameworks. This was followed by a comparison across the contexts to identify particularly illuminating case studies. Particular attention was paid to variations and similarities between and within contexts especially in terms of the response measures adopted and their outcomes [33]. Themes from the two settings were later triangulated. Emerging findings were discussed among the authors and the wider research team in regular meetings, with feedback integrated into the paper. Three case studies were developed to highlight key adaptation measures and their impact in the different settings [34]. These include the expansion in the use of technology in primary healthcare service delivery in Merseyside (Fig. 2); community engagement to promote continued use of maternal and child health services in Liberia (Fig. 3); and disruptions to breast cancer services in Merseyside (Fig. 4). We applied the people-centred health system framework to interpret the data, develop the themes and frame the cross-context lessons described in the Results and Discussion sections.

## Results

Key findings are described in themes below. These are presented alongside illustrative quotes from the data and relevant case studies.

### Digital technology facilitated the provision of essential health services but hindered access to and quality of care among vulnerable populations

As exemplified in Fig. 2, COVID-19 led to the rapid adoption and expansion of digital technology in health service delivery in both settings. In Merseyside, respondents noted virtual consultation enabled continued access to care among clinically vulnerable patients, such as those on cancer treatment. Most were advised to shield at home and would have struggled to obtain needed care without such an intervention. Virtual consultation was seen as convenient and may have minimised barriers to care among younger people, as this quote suggests: *“… lots of patients, especially the youth, are happier having that [video and telephone consultation] and may be accessing care more because if they’ve got a long way to travel, then they don’t need to travel, they don’t need to worry about their parking. They don’t need to worry about having to sit in a waiting room for an hour”* (Hospital consultant, Merseyside). Many clinicians perceived virtual delivery to be as effective as in-person delivery, with the added benefit of greater convenience to patients, for example:: “*virtual consultation has highlighted different ways of working, and probably more efficient ways of working sometimes as well … In a lot of cases, you can gain as much as 90% of your diagnosis from [patient] history, it’s less about the examination.”* (Health worker in community, Merseyside). Some health workers in the community reported telephone triage systems enabled them to efficiently allocate scarce resources to patients who needed care most.

Despite the perceived benefits, respondents in Merseyside noted elderly patients; people with mental health conditions or learning disabilities; people who were homeless or had history of alcohol or substance misuse were disproportionately affected by the transition to telemedicine due to limited capacity to use a smart phone, as illustrated by this clinician: “*we’ve been told to use e-consultations as much as possible. But we’ve got a lot of patients who haven’t got a computer…they are of a different generation and struggled to access care during the pandemic….”* (Health worker in community, Merseyside). Several clinicians complained virtual consultation and reviews did not permit optimal patient assessment and led to misdiagnosis and mistreatment, especially among persons with learning disabilities and persons with impaired vision and cognitive function. They noted effective assessment required considering physical attributes and interactions with carers and the environment. Another widely identified challenge was the ability to build rapport. Even senior primary care staff acknowledged a fear of missing important aspects of patient diagnosis and care.

*“… I think you lose an element of connection - ability with people to ask questions, to challenge you, to ask for more information. And there is a level of nuance that you don’t get.”* (Hospital consultant, Merseyside)

In Liberia, rather than patient engagement, virtual communication tools (in the form of social media) were employed to supervise and engage with community health workers to ensure continued provision of essential services in rural communities amid lockdown and travel restrictions. Such communication tools (e.g., Zoom, Microsoft Teams) were widely deployed within hospitals in Merseyside to facilitate multi-disciplinary collaborative working among healthcare professionals to enhance patient care and staff wellbeing.

### Routine service reductions and cancellations disproportionately affected socially disadvantaged groups and older populations

Initial COVID-19 response measures in both study settings involved diverting resources meant for routine service delivery, notably personnel, equipment, and medical supplies. Respondents were of the view that reallocation was necessary to prevent hospitals becoming overwhelmed by COVID-19 cases, and it was sometimes more efficient. However, many elective services in primary and secondary care were reported to have been discontinued due to capacity constraints caused by the reallocation, resulting in long waiting times, delays in critical investigations and treatment, and compromising quality of care (Fig. 4). Health workers reported an increase in patients presenting with advanced forms of health conditions in the months following the service cancelations. In Merseyside, respondents felt blanket discontinuation of elective services was unhelpful and suggested a more nuanced approach to balance long- and short-term risks be adopted. Further, they recommended that prior planning along with clear and transparent protocols on how services should be suspended and restored. Admission requirements for intensive care units (ICU) in Merseyside were adjusted upwards to escalate capacity for COVID-19 care which denied several non-COVID-19 patients’ access to needed care. Some older patients and people with learning disabilities were perceived to have been disproportionately affected by the rationing during the first wave because access decisions were often made to favour patients with a higher chance of survival. Clinicians expressed difficulty in making escalation decisions and a lack of adequate formal guidelines to support them. Escalation decisions were further compounded by restrictions to family visitation to hospitals.

Meanwhile, Liberia placed strong emphasis on the need for continuation of essential health services (Fig. 3). Community health volunteers involved in community outreach for routine services were deployed to help with contact tracing and awareness raising on COVID-19, diminishing access to neglected tropical diseases (NTDs) and maternal and child health services. Hospital staff were having to split their time between screening COVID-19 patients and providing routine care, leading to long-waits and staff fatigue. National reference laboratories were repurposed for COVID-19 testing, limiting investigations needed by primary and secondary care providers.

*“Some of the community health workers have been positioned to immigration check points to provide temperature checking and all of that. Those are volunteers that are supposed to be in the communities providing NTDs services…. That has a direct negative impact on the NTDs services at the community.”* (National decision-maker, Liberia)

Service cancellations in Merseyside were noted to have disproportionately affected socially disadvantaged groups with pre-existing difficulties with healthcare access, such as ethnic minorities, refugees, and low-income households, in comparison to other groups who could afford private care as an alternative. In Liberia, respondents identified rural residents, people living with severe physical disabilities, children under 5-years of age, pregnant women, and people with chronic disease as those who were most affected by the disruptions in service delivery.

Several measures were identified for improving equitable delivery of routine services in the context of the capacity constraints. In both contexts, cross sectoral collaboration was felt to have contributed to lessening pressures on routine service capacity. Partnerships formed between NHS trusts and private sector organisations facilitated the provision of cancer operations including for patients who could not afford private care following the service closures. In Liberia, engagement with the media helped to counteract misinformation about COVID-19 and promote service utilisation. Social prescribing and community-based service delivery were implemented to ensure the continuation of essential routine services while reducing pressure in hospitals for COVID-19 care in Merseyside. This was noted to be particularly useful for older people and clinically vulnerable patients who could not visit facilities to access care as well as low socio-economic patients whose access were curtailed by disruptions in transport and shift to telemedicine.

### Significant decline in utilisation of essential health care services by the most vulnerable

Participants in both contexts reported huge reductions in the uptake of emergency and elective services. Uptake was particularly low among people with high healthcare needs: the elderly, pregnant people, and people with ongoing chronic illness. They were concerned about contracting COVID-19 at face-to-face appointments. In Merseyside, interviewees felt patients were not coming for needed consultations as the media and government were perpetuating the message that services were unavailable. Government ‘stay at home’ messages in the UK may have been perceived literally, while disruptions in local transport services further constrained physical access. In Liberia, respondents reported a decline in treatment adherence and an increase in self-medication with many pregnant women staying at home and using non-professional care (e.g., traditional birth attendants) leading to increased adverse pregnancy outcomes (Fig. 3). Many patients feared misdiagnosis with COVID-19 and subsequent quarantine if they visited a health facility. Uptake of child immunisation was hugely impacted by “*widespread misperception that the vaccines were contaminated with COVID”* (County decision-maker, Liberia). Respondents reported patients presenting with advanced illness and feared that the delay would lead to excess deaths.

*“During the peak, nobody was coming to hospital with anything. Nobody turned up with strokes to the emergency department. … I think there’s going to be a lot of missed cancer diagnoses…. these are really going to negatively affect people if they couldn’t have their chemotherapy and their cancer has now progressed.”* (Hospital health worker, Merseyside)

*“…for the last two weeks or so some of the deaths we have had, they are not COVID-19 related death, but people thought that they had COVID-19 so they stay away and by the time they present them to the hospital some of them end up having renal failure and they end up dying of heart attack.”* (County decision-maker, Liberia)

In Liberia, as highlighted in Fig. 3, health system actors placed strong emphasis on working alongside community leaders to dispel misperceptions about COVID-19 and to reverse the trend in service utilisation. The need for transparent communication, culturally sensitive messaging, and dignity and respect for service users was highlighted by participants in both settings.

### Physical distancing enhanced safety, limited investigation and care, and diminished psychosocial support for patients

Respondents across both contexts highlighted the positive effects of the infection control measures, such as PPE, physical distancing, and hand and face hygiene, in minimising the spread of COVID-19 and other nosocomial infections. In Merseyside, hospital-based routine services were divided into designated areas according to a traffic light system originally developed for the EVD response, with patients cohorted to minimise interaction between those with COVID-19 and those without COVID-19. Many respondents identified such a system to have streamlined internal patient flow and prevented COVID-19 infections.

In Merseyside, professionals felt restrictions on physical contact due to PPE, which improved patient safety but inhibited patient interaction and quality of care. Many reported that the wearing of gowns, visors and masks hindered their ability to examine patients, build rapport and be heard and seen. This particularly affected the elderly and those with visual and hearing impairment. Patient education, conducted virtually or whilst wearing PPE, limited the kind of conversations that health workers were able to have with patients and were even more difficult among patients with hearing and speech impairment. Further, respondents noted that the lack of face-to-face interaction coupled with the ban on family visitation to hospitals denied patients psychosocial support, stunting their recoveries, especially among elderly who had less access to smart phones and face time (See Fig. 4). In Liberia, the introduction of mandatory face covering in health facilities prevented many low-income patients from seeking healthcare as they could not afford them. Hospitals in Merseyside introduced virtual family contact via Facetime early on, which received positive feedback from both providers and patients.

### Reduced morale, wellbeing, and performance among health workers

The effects of the COVID-19 response on the morale and wellbeing of health workers were widely discussed by respondents across both settings and noted to have affected staff performance in the delivery of quality routine health services. The redeployment of routine health staff was linked with increased workload in both settings, with evidence of staff burnout, fatigue and poor mental health reported. Staff satisfaction and morale in Merseyside was diminished by the lack of face-to-face contact with patients and colleagues which negatively affected performance. Some providers tried to alleviate these through virtual therapy with a psychologist as well as online quizzes and simultaneous team tea-breaks (see Fig. 2). Increased staff collaboration and teamwork were noted and linked to enhance staff mental health in Merseyside. In both settings, staff performance was affected by shortages of personal protective equipment (PPE), with most frontline health workers facing decisions about their personal safety when treating patients.

*“There were some health clinicians that were out of fear either turning patients away or refusing to see patients. Some will just give prescriptions and might not do proper examination because of the fear factors and the unavailability of the requisite PPE.”* (National decision-maker, Liberia)

In Merseyside, redeployment and remote working was noted to have disrupted staff supervision and support, with many junior health workers having to work in isolation without adequate support and oversight. Similarly, in Liberia, in-person supervision of community health workers was pared back due to social distancing and shortage of PPE. Although social media was later employed to provide virtual supervision, respondents noted this may have had a negative impact on the quality of routine services, especially in rural areas, where connectivity was challenging.

### Centralised decision-making hindered responsive health service delivery at local level, undermining a people-centred equitable health service delivery

Respondents across both contexts felt the limited influence of local actors in health decision-making hampered the development of timely solutions to routine health service delivery and exacerbated waiting lists and health equity issues. At the start of the pandemic, several health decisions about routine services, such as the cancelation of elective care in Merseyside and the deployment of health staff to COVID-19 services in both settings, were centralised top-down directives passed down to the local level for implementation. Participation of community actors in priority setting and response decisions were reported to be limited in both contexts. In Merseyside, respondents perceived centralised directives were oriented towards achieving political objectives and did not reflect local realities. These often led to friction between local leadership and frontline staff who wanted scope to influence the decisions. Many reported that they felt under-valued and unmotivated due to their limited participation in health decisions. However, on occasions where staff were given the opportunity to influence local health decisions, specialised committees harnessed staff expertise and appropriate solutions to effective local service delivery were developed; there was greater staff participation; and participants (staff) reported greater job satisfaction. In Liberia, working with community members and local governance structures enabled health system actors to address community misperceptions about childhood vaccination, improve confidence in the health systems and to promote uptake of routine health services during the pandemic (Fig. 3).

### Lack of advance pandemic preparedness hindered effective routine service delivery

Participants in Merseyside noted that local response in routine service delivery was severely hampered by a lack of advance pandemic preparedness. Although some NHS hospitals had a pandemic plan, it was based on an influenza pandemic, and not regularly updated. A lack of relevant local information to inform modelling work and other response strategies in Merseyside was widely reported. As a result, there was shortage of medical supplies such as PPE, human resources and “*confusion over what and how healthcare services should be provide and prioritised at the early stages of the pandemic*” (Hospital health worker, Merseyside). Respondents noted that the capacity shortfalls were compounded by historic underfunding of the health services. In Liberia, learning from experience of EVD epidemic, health system actors recognised the need for early preparedness prior to the COVID-19 pandemic and steps were taken to support the continuation of routine services. However, these efforts were undermined by funding constraints and existing capacity gaps in the health service.

## Discussion

Overall, we found early response measures implemented to tackle the COVID-19 pandemic in Liberia and Merseyside had mixed effects on the equitable delivery of routine essential health services, impacting both the demand- and supply-side of access and quality of care. The adoption of digital technology in health service delivery enabled continued access to vital health services in both contexts but was associated with sub-optimal diagnosis and care among elderly and people living with disabilities (including mental health) in Merseyside. Reallocation of existing health resources to COVID-19 care diminished the capacity for routine health service delivery in both settings, with socially vulnerable populations (e.g., low-income mothers in rural communities in Liberia; ethnic minorities and homeless people in Merseyside) disproportionately affected by delayed diagnosis and treatment for critical health conditions. Redeployment of health workers responsible for routine service delivery increased staff workload and affected their mental health and performance in both contexts. While limited local autonomy and participation in COVID-19 response hindered local innovations and entrepreneurship and alignment to local priorities in Merseyside, in Liberia, emphasis on greater community engagement in response planning improved the utilisation of non-COVID-19 essential health services during the pandemic.

Our findings suggest that although health system measures introduced in the wake of the COVID-19 pandemic in UK and Liberia may have been useful in dealing with the increased demand for care from the pandemic, they led to unintended adverse impact on access and use of critical non-COVID-19 health services. This is consistent with growing evidence of COVID-19 measures disrupting essential health services [35–37]. Pujolar et al. conducted a review and found general reduction in service utilisation in the early stages of the pandemic due to resource constraints, associated lockdown and redeployment of health service resources [21]. In Uganda, Tumwesigye et al. found that lockdown measures introduced in the wake of the pandemic greatly reduced access to and utilization of OPD, malaria treatment, immunisation, and antenatal care services, along with increased excess deaths from TB mortality [38]. Similarly, a survey conducted by the WHO showed that COVID-19 related lockdown and staff reassignment significantly disrupted health services nearly half of countries, particularly affecting non-communicable disease services [13]. In our study, we noted the pandemic response measures may have affected equity and quality of care as much as access. In both contexts, people widely noted to be underserved by the health system were also those disproportionately impacted by the negative consequences of the response measures.

The inequities of the COVID-19 response measures in our study were in part linked to pre-existing disadvantages of vulnerable groups limiting their capability to adapt to the constraints to healthcare imposed by the pandemic [39]. The highly centralised COVID-19 measures adopted in both settings often failed to account for pre-existing barriers to healthcare among socially vulnerable groups and compounded established disadvantages. Further, health systems of both contexts were constrained by pre-existing capacity shortfalls, linked with historic underfunding and, in some cases, weak pandemic preparedness. Faced with a declining health spending resulting partly from cuts to donor funding [40], civil war (1989–2003), and the 2014–2015 EVD epidemic, the Liberia health system experienced vulnerabilities in health infrastructure and human resources, which undermined COVID-19 response efforts. In the UK, years of austerity and funding cuts to the health service prior to COVID-19 had led to serious workforce shortages, especially nursing staff who were particularly needed to provide care during the pandemic in Merseyside and other counties [41]. Moreover, inadequate pandemic preparedness meant that not enough buffer stock of medical supplies and health staff were available to provide the needed surge capacity to maintain routine service delivery in the UK [8].

Findings from the current study highlight important lessons for optimal delivery of routine essential services in the ongoing COVID-19 response and future shocks. These are aligned with the people-centred approach and have been outlined in two separate policy briefs focusing on Liberia [42] and Merseyside [43]. Notably, they suggest the need for pandemic responses to be informed by equity considerations, including tackling immediate pandemic-related and established structural barriers to care [44]. Current COVID-19 responses should aim at reducing financial barriers to care and public distrust as well as improving access to diagnostic testing, PPE, vaccines and other social determinants of health such as housing and secured jobs. Harnessing the equity potential of telemedicine would require closing the digital divide in healthcare by improving digital health literacy and expanding access to relevant technologies among vulnerable populations alongside strengthening the technological infrastructure and skills of healthcare providers [45, 46]. Proactive early engagement and communication with communities based on culturally sensitive approaches emerged as a key component of a people-centred equitable health service delivery. Clear and transparent communication is critical for building trust among health system stakeholder necessary for improved support for pandemic response and delivery of essential health services [9]. As pandemics are likely to create misinformation and changes to existing health services, effective community engagement that cultivates new and existing relationships is central to dispelling myths, raising awareness about services, and learning about community health needs and preferences to inform the delivery of responsive services [44]. Engaging with and promoting the participation of socially vulnerable groups and people with high health needs in response planning is particularly important to developing appropriate actions that address longstanding health inequalities. This should be embedded within a wider framework that engender local autonomy and participatory leadership to develop responsive solutions and build appropriate local partnerships to promote service delivery [47]. Local pandemic response decisions should be informed by robust health data based on reliable tracking of service utilization and health outcomes, including the equity and quality impact of new interventions introduced in response to the pandemic [48].

Our findings underscore the need to strengthen the building blocks of the health system in normal times to create surge capacity and enable ongoing health service delivery during a crisis [49]. Governments need to explore opportunities for increasing funding for routine services between shocks, including innovative approaches to domestic resource mobilisation and the recalibration of health allocations of national budgets. Increased donor funding on health is needed to enable low-income countries with fragile health systems to strengthen their resilience between shocks. Addressing the persistent capacity gaps necessitates greater collaboration between partners within and outside of the health system [44, 47]. Public-private partnerships may be particularly useful in addressing the COVID-19 legacy of increased backlog of routine services in the UK [50] and supporting sustainable medical supplies in remote communities in Liberia.

Further, we identified that improving the capacity and wellbeing of health staff is critical to engendering health system resilience to assure delivery of essential health services. There is the need to ensure the availability of sufficient staff in normal times so that in crisis these are not overstretched. While staff redeployment may be necessary to deal with staff shortfalls in the immediate pandemic period, the process should be driven by judicious planning and workload management backed by data on staffing needs to minimise potential unintended consequences in other essential service areas. Improving job satisfaction among health workers through financial and non-financial incentives is essential to boosting morale and enhanced performance [50]. With pandemics requiring the rationing of services, training, and guidance on new roles and on ethical decision-making for staff is imperative. The impact of COVID-19 on the wellbeing and mental health of health workers both in the short and longer term should not be underestimated. Wider action on the workforce must be underpinned by better support for staff wellbeing, including access to psychological support, compassionate leadership, and supportive working environments [9, 51].

Consistent with Cascini et al., advance pandemic preparedness based on participatory and inclusive planning process, along with frequently updated locally informed guidelines and intersectoral collaboration are essential for assuring effective health services during pandemic [9].

## Limitations

The cross-sectional qualitative study design may limit the generalisability of our findings. However, the purposive sampling and data saturation techniques employed to select the participants enriched the study data and enhanced the validity of the findings. The comparative approach of the study involving participants being selected across different levels of the health system in the study countries improved the breadth of the data but made comparison difficult. The different level of participants used in the two study settings (national and county level decision makers in Liberia compared to mainly frontline health workers in UK) may have resulted in some of the differences in findings related to differing perspectives. Further, not including a larger number and range of participants from sub-national health systems levels in Liberia may have limited the depth of understanding about the impact of COVID-19 response at the local level in the country. The study was carried out at a single point in time and at an early stage of the pandemic when response measures where only starting to be introduced. Since then, the pandemic and related response measures have continued to evolve and so would be their impacts. An impact analysis of the COVID-19 response on equity and quality of routine services from the perspective of service users would strategically complement this data and analysis.

## Conclusion

This analysis of the impact of the COVID-19 response measures on routine services shows that while they sometimes improved safety and ensured continued access to vital health services during the pandemic, they disrupted equitable access to many routine essential services, with socially vulnerable groups disproportionately affected. Pre-existing health system capacity and social inequalities shape the ability to maintain equitable access to quality routine services during COVID-19. Evidence from this research can be useful to inform pandemic response planning to ensure optimal delivery of essential health services during public health emergencies. Ongoing COVID-19 responses and future shocks should prioritise addressing both the immediate and structural barriers to care; evidence-based practice and decision-making; and robust engagement with communities and patients, leveraging culturally sensitive and inclusive approaches. It is essential to ensure adequate funding and investment in the building blocks of the health system in normal times. Further, there is the need for greater autonomy for local decision-making coupled with inclusive and participatory leadership that promote the involvement of staff and community leaders in local decisions on health services.

## Supplementary Information

**Additional file 1.**

## Data Availability

Data analysed for this manuscript are available from the corresponding author on reasonable request.

## Abbreviations

EVD: Ebola virus disease
ICU: Intensive care unit
NHS: National health service
NPI: Non-pharmaceutical intervention
NTD: Neglected tropical disease
PPE: Personal protective equipment
WHO: World Health Organisation
