# Supplementary material for: Perceived impacts of COVID-19 responses on routine health service delivery in Liberia and UK: cross-country lessons for resilient health systems for equitable service delivery during pandemics
Source: BMC Health Serv Res. 2023 Mar 29;23:304. doi: 10.1186/s12913-023-09162-8 (PMC10057690; doi:10.1186/s12913-023-09162-8)
Supplement: Supplementary file 1 — Additional file 1. [file 12913_2023_9162_MOESM1_ESM.docx]

## **Key Informant Interviews Topic Guide –Merseyside Regional Decision Makers**

**Background**

Please can you tell me your position and how long you have worked in your current role?

**Impact of COVID 19 on Routine Service Delivery**

1. What are defined as essential routine services?
2. How has delivery of these services changed?
3. Which are the main scheduled and unscheduled services affected?
4. How have these services been affected?
5. Describe the changes that have been made?
6. How successful do you feel these changes have been in keeping services going?
7. Have there been any innovations within service delivery, and what have these been?
8. Have these innovations been useful in any way?
9. Have there been any changes that have concerned you? Why?
10. What guidance documents are available to support you in making decisions?
11. What would help to support maintaining routine services?

**Governance and Decision Making**

1. During the current pandemic, what processes can lead to disruption of health services? (Such as health worker illness)
2. How have these been considered in decisions around maintaining routine services?
3. Who is involved in decisions made about which services should or should not be prioritised?
4. How are decisions made about which services should or should not be prioritised?
5. Describe how and who is involved in operationalising decisions?
6. What challenges have you faced in making these decisions?
7. What organisational structures, people or collaborations exist to support decision making?
8. How are decisions being communicated through the system?
9. Who are the decision makers in terms of allocating medical resources in hospitals? Is there an independent committee for it? Do frontline health workers have to make these decisions? Who do you think should be the decision makers? Why?
10. How are changes in service delivery communicated? How can this be improved?

**Human Resource Management**

1. How have you [may be the employer in general] planned for staffing to meet the changing additional workload? Any tools/ guidance from national authorities? Successes and challenges?
2. How have you planned for the increase in staff absence?
3. What are the main sources of additional staffing (e.g. secondment/redeployment, task-shifting, improved productivity, early graduation/students, returnees, volunteers)?  Impact on the wage bill? Successes and challenges?
4. What areas of service are now less well staffed?
5. What are you able to do to retain staff?
6. What have been the successes and challenges with staff retention?
7. What additional skill development have you provided and how?
8. What have been the successes and challenges with skill development?
9. How are you able to support staff so they can continue to work effectively (e.g. communication, occupational safety including PPE, psychosocial support)?
10. What have been the successes and challenges with supporting staff to work effectively?

**Recovery post COVID-19**

32. Are there any COVID-19-related changes to routine health services that you think it would be useful to continue after COVID-19? Which ones and why?

33. What next steps do you believe should be taken now to support the health system to recover post COVID-19?

Thank-you

Do you have any further suggestions for improvements to delivery of routine services?

Any other comments?

##

## **Key Informant Interviews Topic Guide – Merseyside Health Workers**

**Impact of COVID 19 on Routine Essential Service Delivery**

1. Can you tell me about how health service delivery has been affected by the COVID pandemic?
2. Please tell me more about the process for how change to service delivery happens. Do you have any ideas about how this can be improved?
3. How are changes in service delivery communicated? How can this be improved?
4. What do you consider to be routine essential health services?
5. [If not already discussed] Can you tell me about how routine essential service delivery has been affected by the COVID pandemic?
6. How have routine essential services been modified or adapted? Can you provide examples of any innovations?
7. What have been the strengths and challenges with these changes?
8. How should these changes be evaluated? What indicators should be used?
9. Which services would you say have been most impacted so far? (e.g. hospital based, community care, disease specific services, etc) Why?
10. How has referral changed from primary to secondary care for [select option based on participants’ role] maternal health care/ a patient with a chronic disease already known to the hospital team/ a patient with a suspected cancer diagnosis?
11. How has quality of your service been impacted through these changes?
12. Are you documenting these changes? How will you evaluate the impact of these changes?
13. What is worrying you most about your service now?
14. Which services would you envisage will be most impacted moving forwards as the pandemic progresses? (e.g. hospital based, community care, disease specific services, etc) Why?

Who do you think are the people most impacted by the changes in routine service delivery? Would you say that patients with specific socio-demographic characteristics are more impacted by service disruption/ distortion than others? Why? (e.g. gender, dis/ability; rural/urban; wealth; geographic regions; age etc)

What can be done to ensure that these patients can still use health services when they need them?

1. How is quality of care being maintained during this pandemic? And do you have any suggestions to improve quality?

**Ethics and Decision Making**

1. Have you encountered any issues which you found troubling since the start of the COVID-19 pandemic?
2. Would you be willing to tell me more about these issues?
3. What is the impact of these issues on you as a health worker?
4. What would be helpful to support you in dealing with these issues?
5. Do you know of any ethical guidelines in place to guide you as you make difficult decisions during this time? What are these?
6. Have you ever approached the trust board?
7. Have you been involved with making decisions about the changes to health services since the COVID-19 pandemic?
8. What was your role in making these decisions? How were these decisions made?
9. How are these ethical guidelines operationalised? Are they useful? Do you think that as a frontline health worker you have enough understanding about these ethical guidelines? Why?
10. How are decisions communicated?
11. Who are the decision makers in terms of allocating medical resources in hospitals? Are you involved in making these decisions? Do you want to be involved in these decisions? Why?

**Human Resource Management**

1. How has your role changed since the start of the COVID-19 pandemic?
2. What have been the successes and challenges with how your role has changed as part of the COVID-19 response?
3. Is there anything about your role that concerns you? What?
   1. Probe working outside are of expertise
   2. No indemnity if make an error
   3. Communication about working across disciplines
4. What preparation for the changes to your role have you had and how was it delivered (skills - key ones, psychological support)?
   1. Probe PPE training
   2. COVID clinical training
   3. Support mechanisms
   4. Team formation
5. What have been the successes and challenges with the preparation you received for these changes?
6. What kind of support to do your job (e.g. communication, occupational safety including PPE, psychosocial support) are you receiving from your team/manager/employer?
7. What have been the successes and challenges with the support you have received to do your role?

**Recovery post COVID-19**

36. Are there any COVID-19-related changes or innovations to routine health services that you think it would be useful to continue after COVID-19? Which ones and why?

37. What next steps do you believe should be taken now to support the health system to recover post COVID-19?

38. What is worrying you most as the response moves forward?

Thank-you

Do you have any further suggestions for improvements to delivery of routine services?

Any other comments?

## **Key Informant Interviews Topic Guide –Merseyside Laboratory and Blood Transfusion Staff**

**Governance and Decision Making - Relating Directly to COVID-19**

1. What has been the decision-making process for the laboratory’s response to COVID-19 testing services and when did discussions start around re-adjusting services for COVID-19?

2. Who held overall responsibility for how COVID-19 testing was going to be conducted at LCL?

3. In addition to PHE, have the Liverpool Clinical Laboratory services worked closely/ collaborated with any other external partners for COVID-19 testing? If so whom and in what capacity?

**Governance and Decision Making - Relating to Maintaining Routine Service Delivery**

4. How are decisions made about which services should or should not be prioritised; which ones were considered to be essential and why? Who is involved in this decision making? How were these decisions communicated?

5. What guidance documents were most useful to you in making these decisions? In what way were they useful?

6. What key challenges have you faced in making these decisions? Do you have any support needs here?

**Impact of COVID-19 on Routine Laboratory Service Delivery**

7. Can you tell me about how routine clinical laboratory service delivery has been affected by the COVID pandemic?

COVID-19 Testing service specific

8. How did the laboratories adapt to scale up COVID-19 testing? (analysers, staff capacity, staff training, standard operating procedures, risk assessments)

9. What challenges did the laboratory face when implementing COVID-19 testing? How were they overcome? What worked well? (e.g. resources, human resource, process change, governance, culture, leadership etc)

10. Which routine services would you envisage will be most impacted moving forwards? (e.g. hospital based-testing, disease specific services, etc) Why?

**Recovery post COVID-19**

11. Are there any COVID-19-related changes to the laboratory service that you think it would be useful to continue after COVID-19? Which ones and why?

12. What next steps do you believe should be taken now to support the laboratory system to recover post COVID-19?

13. Are there any changes/ innovations introduced in response to COVID-19 changes which you think should be continued? Why?

Thank you

Do you have any questions for me? Resources (re labs) link https://www.rcpath.org/uploads/assets/90111431-8aca-4614-b06633d07e2a3dd9/Guidance-and-SOP-COVID-19-Testing-NHS-Laboratories.pdf

## **Key Informant Interviews Topic Guide –Liberia MOH staff**

**Background**

Please can you tell me your position and how long you have worked in your current role?

**Responses to Shock and the General Health System**

1. How is the current shock the health system is experiencing similar or different to those you have experienced before?
2. What are the key learnings from previous shocks that you think are supporting the system to respond now?
3. What are the key learnings from previous shocks that you think are not being used to support the system to respond now?
4. How do you think routine health systems functions are being impacted by the current crisis?
5. What do you think could be done to support continuation of routine services? How is this informed or shaped by learnings from during the Ebola period?
6. What key steps do you believe should be taken now to support the health system to recover post crisis?
7. What policy or guidelines are supporting with the current response? What additional guidelines or policies could be helpful?
8. What needs to be strengthened to have a resilient health system for future crisis?

**Service Specific Impacts**

1. Can you tell me about how service delivery within *your programme/section (adapt to include name of section depending on who talking too)* has been affected by the COVID pandemic?
2. How have your routine services been modified or adapted? Which components of your service do you view as essential? Why?
3. Which of your services would you say have been most impacted so far? Why?
4. Which services would you envisage will be most impacted moving forwards? Why?
5. Which specific sub-populations is routine care most impacted for? (e.g gender, dis/ability, rural/urban; wealth; geographic regions; age etc)
6. How is quality of care being maintained?
7. What key steps do you believe should be taken now to support your service to recover post crisis?
8. Have there been any innovations within service delivery, and have they been useful in any way?
9. Has there been any changes that have concerned you?

**Service and System Impacts: Governance and Decision Making**

1. How are decisions made about which services should or should not be prioritised? (prompt for in relation to their specific service and also in relation to general health system)
2. Who is involved in this decision making and what are the processes?
3. What do you think are the key ethical impacts of making these decisions? What ethical guidelines are currently in place and important in decision making during this period?
4. Describe how and who is involved in operationalising decisions?
5. What challenges have you faced in making these decisions?
6. What organisational structures, people or collaborations exist to support decision making? Ask specifically about the influence of donors, funding regulations and flows.
7. How are decisions being communicated through the system?
8. What guidance documents are available to support you in making decisions?
9. What would help to support maintaining routine services?

**Human Resource Management**

1. How have you planned for staffing to meet the changing additional workload? Any tools/ guidance from the human resource section? Successes and challenges?
2. What are the main sources of additional staffing (e.g. secondment/redeployment, task-shifting, improved productivity, early graduation/students, returnees, volunteers)? Impact on the wage bill? Successes and challenges?
3. What areas of service are now struggling with staffing?
4. What are you able to do to retain staff? Successes and challenges?
5. What impact did/is down-sizing of “non-essential staff’ have on your programme during the crisis?
6. What additional skill development have you provided and how? Successes and challenges?
7. How are you able to support staff so they can continue to work effectively (e.g. communication, occupational safety inc. PPE, psychosocial support)? Successes and challenges?

**Closing Questions**

1. What are your three recommendations would you make to improve or maintain the resilience of the Liberian health system during this period?
2. What are your three recommendations would you make post crisis to ensure the return to routine function of the health system as effectively as possible?

Thank-you

Any other comments?
